# „Mild-behavioral-impairment“-Checkliste: Englisch-deutsche Übersetzung und Beurteilung der Anwendbarkeit in klinischer Praxis

**DOI:** 10.1007/s00391-023-02200-4

**Published:** 2023-06-26

**Authors:** Pauline Dibbern, Jennifer Horsch, Julia Fiegl, Linda Eckl, Tamara Finger, Lisa Diermeier, Markus Deppe, Stephan Schiekofer, Berthold Langguth, Zahinoor Ismail, Filip Barinka

**Affiliations:** 1 Zentrum für Altersmedizin der Klinik und Poliklinik für Psychiatrie und Psychotherapie, Universität Regensburg, Regensburg, Deutschland; 2 Klinik für Innere Medizin, Krankenhaus Barmherzige Brüder St. Barbara, Schwandorf, Deutschland; 3 Klinik für Allgemeine Innere Medizin und Geriatrie, Krankenhaus Barmherzige Brüder Regensburg, Regensburg, Deutschland; 4 Zentrum für Psychiatrie Cham der Klinik und Poliklinik für Psychiatrie und Psychotherapie, Universität Regensburg, Cham, Deutschland; 5 Klinik für Psychiatrie und Psychotherapie, Bezirksklinikum Mainkofen, Deggendorf, Germany; 6 Fakultät für Medizin, Lehrstuhl für Geriatrie, Sigmund Freud PrivatUniversität, Wien, Österreich; 7 Zentrale Aufnahme und Psychiatrische Institutsambulanz der Klinik und Poliklinik für Psychiatrie und Psychotherapie, Universität Regensburg, Regensburg, Deutschland; 8 Zentrum für Allgemeinpsychiatrie (Zentrum II) der Klinik und Poliklinik für Psychiatrie und Psychotherapie, Universität Regensburg, Regensburg, Deutschland; 9 Department of Psychiatry, Hotchkiss Brain Institute, University of Calgary, Calgary, Kanada; 10 Klinik und Poliklinik für Neurologie, Universität Regensburg, Regensburg, Deutschland; 11 Dr. Filip Barinka, Altersneurologie und Gedächtnis-Sprechstunde, Bürglistrasse 29, 8002 Zürich, Switzerland

**Keywords:** Leichte Verhaltensbeeinträchtigung, Demenz, Früherkennung, Übersetzung, Machbarkeitsstudie, Geriatric psychiatry, Dementia, Early detection, Translation, Feasibility study

## Abstract

**Hintergrund:**

Das Syndrom einer leichten Verhaltensbeeinträchtigung („mild behavioral impairment syndrome“, MBI) ist definiert durch das Auftreten anhaltender neuropsychiatrischer Symptome im Alter. Die Mild-behavioral-impairment-Checkliste (MBI-C) dient der Erfassung von persistierenden neuropsychiatrischen Symptomen, welche die Präsenz des MBI definieren.

**Ziel:**

Erarbeitung einer deutschsprachigen Version der MBI‑C und Beurteilung der klinischen Anwendbarkeit.

**Material und Methoden:**

Im Austausch mit dem federführenden Autor der englischen Originalversion wurde eine deutsche Version erstellt. Die Praktikabilität der Anwendung wurde im Rahmen einer Anwendbarkeitsstudie an einer Kohorte von 21 stationären alterspsychiatrischen Patienten überprüft. Dabei wurden die Compliance der Patienten, die Verständlichkeit, der Zeitaufwand, das Vorgehen bei der Auswertung und die Unterschiede zwischen den Angaben der Patienten und der Angehörigen beurteilt.

**Ergebnisse:**

Die erstellte Übersetzung der MBI‑C gilt als offizielle deutsche Version und kann auf https://mbitest.org heruntergeladen werden. Alle Patienten beantworteten alle 34 Fragen vollständig, die Verständlichkeit zeigte sich als sehr gut, der durchschnittliche Zeitaufwand lag bei 16 min. Es zeigten sich z. T. bedeutsame Unterschiede zwischen den Angaben der Patienten und der Angehörigen.

**Diskussion:**

Das MBI kann bei einem Teil der Personen mit neurodegenerativer demenzieller Erkrankung das ansonsten präsymptomatische Stadium markieren. Die MBI‑C könnte somit bei der Früherkennung von neurodegenerativen Demenzen helfen. Diese Hypothese kann mithilfe der hier präsentierten sprachlich lokalisierten Version der MBI‑C auch im deutschsprachigen Raum zukünftig überprüft werden.

**Zusatzmaterial online:**

Zusätzliche Informationen sind in der Online-Version dieses Artikels (10.1007/s00391-023-02200-4) enthalten.

## Abstract (en)

**Background:**

The mild behavioral impairment (MBI) syndrome is defined by the emergence in later life of persistent neuropsychiatric symptoms. The MBI checklist (MBI-C) can be used for systematic detection and documentation of such symptoms.

**Objective:**

Development of a German version of the MBI‑C and assessment of its application in a clinical setting.

**Material and methods:**

The MBI‑C was translated from English into German in collaboration with the main author of the original version, and its practical application was then tested on a study population (*n* = 21) in a gerontopsychiatric inpatient clinic. Patient compliance, understanding of questions, time effort, evaluation procedure and possible discrepancy between patient and family member evaluations were assessed.

**Results:**

The German translation of the original MBI‑C obtained certification as an official version and can be downloaded at https://mbitest.org. All 34 questions were fully completed by the study population, the level of understanding of questions was good, with the mean time effort being 16 min. In some cases, significant differences between patients’ and family members’ responses were found.

**Discussion:**

The presence of MBI may indicate the development of an otherwise presymptomatic neurodegenerative dementia syndrome. Hence, the MBI‑C could aid in the early detection of neurodegenerative dementia. By means of the translated version of the MBI‑C presented in this study, this hypothesis can now be tested in German-speaking countries.

## Hintergrund und Fragestellung

Die Frühphase einer neurodegenerativen demenziellen Erkrankung, v. a. der Alzheimer-Krankheit (AD), ist häufig durch das Syndrom einer leichten kognitiven Beeinträchtigung („mild cognitive impairment“, MCI) markiert [1, 17].

Die kognitiven Defizite bilden aber nur einen Teil des Symptomspektrums bei verschiedenen Demenzarten. Insbesondere bei anderen (non-AD) neurodegenerativen Demenzen, in erster Linie bei der frontotemporalen Demenz (FTD), stehen bereits in der Frühphase vermehrt Verhaltensauffälligkeiten und nichtkognitive psychische Symptome im Vordergrund.

Solche Symptome sind zwar auch bei AD bekannt und werden als „psychische und Verhaltenssymptome“ („behavioral and psychological symptoms of dementia“, BPSD) oder „neuropsychiatrische Symptome“ („neuropsychiatric symptoms“, NPS) bezeichnet. Sie wurden jedoch vorwiegend als Begleiterscheinung erst späterer Krankheitsphasen wahrgenommen.

Diese nichtkognitiven Symptome können aber auch bei AD, zumindest bei einem Teil der Patienten, ein ansonsten noch asymptomatisches Stadium einer demenziellen Entwicklung markieren [2, 7, 12]. So wurde die Übertragung eines ursprünglich für die FTD entwickelten Konzepts einer „leichten Verhaltensbeeinträchtigung“ (Mild behavioral impairment, MBI) auch auf andere Demenzformen diskutiert und definiert ([11, 18]; Tab. 1). Im Gegensatz zu den Begriffen NPS oder BPSD soll das MBI in Analogie zu einem MCI die Aufmerksamkeit auf die klinische Frühphase eines neurodegenerativen Prozesses richten.

**Table 1:** 

1.	Im Alter > 50 Jahre *neu aufgetretene* klar erkennbare Veränderungen im Verhalten oder in der Persönlichkeit des Patienten (beobachtet vom Patienten, von Angehörigen oder von Klinikern), *persistierend oder intermittierend auftretend im Zeitraum >* *6 Monaten*. Die Beeinträchtigung liegt in mindestens einem dieser Bereiche vor: a) Motivationsstörungen b) Affektive Dysregulation c) Impulskontrollstörungen d) Sozial inadäquates Verhalten e) Abnormalitäten der Wahrnehmung oder der Denkinhalte
2.	Die Veränderungen sind von suffizientem Schweregrad, um *zumindest minimale Beeinträchtigung im Alltag* zu verursachen
3.	Die Veränderungen sind nicht als Folge einer aktuellen psychiatrischen oder körperlichen Erkrankung oder als eine (Neben‑)Wirkung von Medikamenten oder Drogen erklärbar
4.	Der Patient erfüllt nicht die diagnostischen Kriterien für das Vorliegen eines demenziellen Syndroms (gleichzeitige Diagnose einer MCI ist möglich)

Die ausführliche Version der diagnostischen Kriterien wurde von Ismail et al. [11] publiziert

*ISTAART* International Society to Advance Alzheimer’s Research and Treatment, *MBI* „mild behavioral impairment“ – leichte Verhaltensbeeinträchtigung, *MCI* „mild cognitive impairment“ – leichte kognitive Beeinträchtigung

Eines der noch ungelösten Probleme ist die Unsicherheit, inwiefern das Vorliegen derartiger Symptome beim individuellen Patienten für eine neurodegenerative Erkrankung indikativ ist oder eher auf eine andere Erkrankung, z. B. eine depressive Episode, hinweist. Auch bleibt es noch großenteils ungeklärt, welche der einzelnen nichtkognitiven Symptome am meisten mit der späteren Konversion in eine manifeste Demenz korrelieren. Eine Klärung derartiger Fragen erfordert eine standardisierte Terminologie sowie ein einheitliches Vorgehen.

Zu diesem Zweck wurde die MBI-Checkliste entwickelt (MBI‑C; [9]). Mit diesem strukturierten Fragebogen können die vorliegenden Symptome erfasst werden. Die Anwendung dieses Instruments in prospektiven klinischen Studien soll helfen, den Stellenwert der einzelnen neuropsychiatrischen Symptome bei der Früherkennung einer neurodegenerativen Demenzentwicklung zu erforschen. Die Checkliste wird international zunehmend angewendet und ihre Validität, insbesondere bei der Früherkennung einer Alzheimer-Krankheit, evaluiert [3–5, 8, 10, 13, 15, 16, 19].

Da die Fragen der MBI‑C in erster Linie direkt durch die untersuchte Person und durch ihre Familienangehörige beantwortet werden, ist die Verwendung einer für den jeweiligen Patienten sprachlich verständlichen Version notwendig. So wurde die Checkliste bis zum jetzigen Zeitpunkt bereits in 14 Sprachen übersetzt. In dieser Arbeit beschreiben wir zum einen die Übersetzung der MBI‑C aus dem englischen Original in die deutsche Sprache, zum anderen die wichtigsten Merkmale des Instruments sowie die Beurteilung der klinischen Anwendbarkeit an einer kleinen Kohorte von gerontopsychiatrischen Patienten.

## Studiendesign und Untersuchungsmethoden

### Aufbau der MBI-Checkliste

Die MBI‑C ist als Fragebogen an den Patienten, einen Familienangehörigen, eine Vertrauensperson oder eine medizinische Bezugsperson gerichtet. Sie besteht aus insgesamt 34 Fragen, unterteilt in 5 Kategorien entsprechend den MBI-Symptombereichen (Tab. 1).

Die Befragten müssen Entscheidungsfragen zum Vorliegen eines Symptoms beantworten. Es ist wichtig, den Befragten klarzumachen, dass nur solche „Auffälligkeiten“ berücksichtigt werden, die in den letzten 5 Jahren neu aufgetreten sind und gleichzeitig (zumindest zeitweise) über einen Zeitraum von mindestens 6 Monaten bestehen. Bei der Bejahung einer Frage wird zusätzlich nach der empfundenen Intensität des vorliegenden Symptoms gefragt und diese in 3 Stufen aufgeteilt:

- Stufe 1 beschreibt eine leichte (bemerkbare, aber nichtsignifikante) Veränderung,
- Stufe 2 eine mittelschwere (signifikante, aber nichtdramatische) Veränderung und
- Stufe 3 eine schwere (ausgeprägte, dramatische) Veränderung.

Für die Auswertung werden die jeweiligen Punktzahlen nach Vervollständigung des Fragebogens aufaddiert.

### Übersetzung

Die deutsche Übersetzung des englischen Originals wurde erstellt, indem 3 unabhängige Übersetzungen angefertigt wurden, die dann im Konsens miteinander abgeglichen und in Bezug auf die verschiedenen Fragen optimiert wurden. Anschließend erfolgte ein weiterer Dialog mit dem Hauptautor der englischen Originalversion (Prof. Zahinoor Ismail, University of Calgary).

### Anwendbarkeitsstudie

Bei der Beurteilung der Anwendbarkeit der MBI‑C in klinischem Alltag wurden folgende Kriterien evaluiert:

- Bereitschaft und Kooperation der Patienten und Angehörigen bei der Durchführung der Untersuchung mithilfe der MBI‑C,
- Verständlichkeit für die Patienten,
- Verständlichkeit für die Angehörigen,
- Zeitaufwand bei Befragung der Patienten,
- Vorgehen bei der Auswertung, Berechnung der Punktzahl,
- Unterschiede in der Bewertung durch die Patienten und durch die Angehörigen.

### Patientenkollektiv

Für den Einschluss in die Studie wurden stationäre Patienten des Zentrums für Altersmedizin der Klinik und Poliklinik für Psychiatrie und Psychotherapie der Universität Regensburg erwogen. Die Einschlusskriterien waren ein Alter über 50 Jahre und die stationäre Aufnahme aufgrund von neu entwickelten (nicht vor dem 50. LJ bestehenden) neuropsychiatrischen Symptomen, welche (ggf. in geringerer Ausprägung) bereits 6 oder mehr Monate bestanden. Die klinischen Aufnahmediagnosen sowie die Entlassungsdiagnosen (Zusatzmaterial online: Supplement Tab. 1) wurden von den behandelnden Ärzten ICD-10-konform gestellt, unabhängig von den weiteren diagnostischen Verfahren der Studie.

Ausgeschlossen wurden Patienten mit einem demenziellen Syndrom unabhängig von dessen Ätiologie. Das Vorliegen eines nach den Kriterien von Albert et al. [1] diagnostizierten MCI stellte kein Ausschlusskriterium dar.

Von 28 für den Einschluss erwogenen Patienten lehnten 7 die Studienteilnahme ab. Somit wurden 21 Patienten in die Studie eingeschlossen (Tab. 2).

**Table 2:** 

Gesamtzahl der Patienten	21
Weiblich:männlich	11:10
Altersdurchschnitt (Jahre)	73
Altersmedian (Jahre)	76
Altersintervall (Jahre)	51-86
MCI (Patientenanzahl)	9

*MCI* „mild cognitive impairment“

Alle Patienten wurden über die klinische Studie ausführlich mündlich und schriftlich aufgeklärt und willigten schriftlich ein, an der Studie teilzunehmen. Die Teilnahme an der Studie hatte bei den Patienten keinen Einfluss auf die Behandlung durch das behandelnde Ärzteteam.

Die klinische Studie in der hier präsentierten Form wurde von der Ethikkommission an der Universität Regensburg bewilligt (Ethikvotum 19-1322-101).

### Diagnostische Verfahren

Die Patienten wurden fachärztlich (psychiatrisch und neurologisch) sowie neuropsychologisch untersucht. Die Indikation für weitere Untersuchungen wurde von dem behandelnden Ärzteteam gestellt, und zwar in strikter Unabhängigkeit von der klinischen Studie.

Für diese Studie wurde die Möglichkeit der Beantwortung der MBI-C-Fragen in der Form eines Patienteninterviews gewählt. Im Anschluss wurde zusätzlich jeweils ein Familienangehöriger des Patienten mit der MBI‑C befragt.

Die Befragung der Patienten wurde während des stationären Aufenthalts persönlich durchgeführt, die Befragung der Familienangehörigen fand, außer in 2 Fällen, telefonisch statt. Bei 5 Patienten konnten wir eine Verlaufsuntersuchung im Rahmen einer ambulanten Einjahreskontrolle (min. 10 Monate bis max. 18 Monate später) durchführen. Bei geringer Relevanz der Befunde für die Anwendbarkeitsbeurteilung werden diese im folgenden Text nicht diskutiert, können aber in der Tab. 3 abgelesen werden.

**Table 3:** 

	Erstuntersuchung						1‑Jahres-Kontrolle			
Patienten-nummer	MCI	MMSE	GDS-15	MBI-Score Befragung Patient	MBI-Score Befragung Angehöriger	Zeitaufwand MCI‑C Patient	MMSE	GDS-15	MBI-Score Befragung Patient	MBI-Score Befragung Angehöriger
1	Nein	30	11	30	–	15 min	30	6	23	–
2	Ja	26	8	27	30	20 min	–	–	–	–
3	Ja	24	11	36	24	10 min	24	9	16	23
4	Nein	27	11	25	–	20 min	–	–	–	–
5	Nein	28	8	9	22	15 min	–	–	–	–
6	Ja	26	2	0	10	5 min	–	–	–	–
7	Nein	28	6	8	46	40 min	–	–	–	–
8	Nein	27	4	7	25	10 min	26	3	3	11
9	Nein	27	5	10	34	15 min	–	–	–	–
10	Nein	27	8	25	35	15 min	–	–	–	–
11	Ja	28	4	15	–	20 min	–	–	–	–
12	Nein	29	0	5	19	15 min	–	–	–	–
13	Nein	25	–	12	–	10 min	–	–	–	–
14	Nein	25	–	14	–	15 min	–	–	–	–
15	Ja	28	3	8	3	15 min	–	–	–	–
16	Nein	30	8	24	41	10 min	–	–	–	–
17	Nein	28	2	7	–	25 min	30	3	9	–
18	Ja	28	6	6	–	25 min	–	–	–	–
19	Ja	26	4	10	–	20 min	–	–	–	–
20	Ja	27	1	1	11	5 min	27	3	2	8
21	Ja	27	3	3	–	15 min	–	–	–	–

*MMSE* Mini-Mental State Examination, *GDS-15* Geriatric Depression Scale-15, *MCI* „mild cognitive impairment“, *MBI* „mild behavioral impairment“, *MBI‑C* „Mild-behavioral-impairment“-Checklist

## Ergebnisse

### Übersetzung

Die hier präsentierte Übersetzung wurde von Dr. Ismail autorisiert, gilt als offizielle Version für deutschsprachige Regionen und ist unter https://mbitest.org/MBI-german.pdf kostenfrei für wissenschaftliche sowie klinische Anwendung abrufbar.

### Anwendbarkeitsstudie

#### Beurteilung der Bereitschaft der Patienten und der Angehörigen zur Durchführung der Untersuchung mithilfe der MBI-Checkliste

Bei den 21 in die Studie eingeschlossenen Patienten konnte die Befragung mithilfe der MBI‑C in voller Länge durchgeführt werden. Bei 2 von diesen Patienten war die Beantwortung des Fragebogens aufgrund von Misstrauen und wahnhaften Symptomen sehr schwierig. Insbesondere bei Fragen zum Symptombereich „Überzeugungen und Sinneswahrnehmungen“ fühlten sich diese Patienten angegriffen und hatten Angst vor Konsequenzen für den weiteren stationären Aufenthalt bei „falscher“ Beantwortung der Fragen.

Familienangehörige von 12 der insgesamt 21 Patienten konnten befragt werden. Bei 9 Patienten, bei welchen kein Angehöriger befragt werden konnte, lag dies in keinem Fall an einer Ablehnung der Angehörigen. In 3 Fällen konnten die Bezugspersonen nicht rechtzeitig erreicht werden. In den restlichen 6 Fällen wollten die Patienten aus verschiedenen dokumentierten Gründen nicht, dass die Familienangehörigen befragt werden.

#### Verständlichkeit für die Patienten

Der Fragebogen zeigte sich bei allen 21 Patienten gut verständlich. Die Anzahl an Nachfragen war sehr variabel und korrelierte nicht mit der jeweiligen Schwere der Symptome der Patienten. Es wurden häufiger Fragen zu der genauen Bedeutung der verschiedenen Intensitätsstufen der Symptome gestellt und der Untersucher nach seiner Einschätzung gefragt.

#### Verständlichkeit für die Angehörigen

Insgesamt sahen die Angehörigen (*n* = 12) der Patienten den Fragebogen als sehr positiv und gaben sich viel Mühe bei der Beantwortung der Fragen. Generell konnte die Checkliste meist flüssiger ausgefüllt werden als mit den Patienten, und es gab keine Probleme mit dem Verständnis der Fragen.

#### Zeitaufwand bei Befragung der Patienten

Der durchschnittliche Zeitaufwand bei Patientenbefragung mittels MBI‑C betrug 16 min (Median 15 min), zeigte sich aber mit einer Spannbreite von 5–40 min sehr variabel.

Die Beurteilung der Intensität des einzelnen Symptoms beanspruchte meist mehr Zeit als die Frage nach deren generellen Existenz.

#### Vorgehen bei der Auswertung und erreichte Punktzahlen

Der Fragebogen wurde wie von Ismail et al. [9] empfohlen ausgewertet.

Die maximal erreichbare Punktzahl nach Addition aller 34 Fragen beträgt 102 Punkte.

Die Frage nach einer Veränderung des Fahrstils (Frage 12) konnte von 6 der 21 Patienten nicht beantwortet werden, da sie z. T. schon einige Jahre nicht mehr Auto fuhren oder nie einen Führerschein besaßen. In solchen Fällen ist die Frage mit 0 Punkten zu versehen.

Der erreichte Mittelwert aller 21 Patienten betrug 13 Punkte (Spannbreite von 0 bis 36 Punkten, Median 10 Punkte; Tab. 3). Bezogen auf die einzelnen Symptombereiche betrug der Mittelwert in der Kategorie „Motivationsstörungen“ 3,7 Punkte, in „affektive Dysregulation“ 5,6 Punkte, in „Impulskontrollstörungen“ 2,5 Punkte, in „sozial inadäquates Verhalten“ 0,4 Punkte und in „Abnormalitäten der Wahrnehmung oder der Denkinhalte“ 1,2 Punkte.

#### Unterschiede bei der Bewertung durch die Patienten und durch die Angehörigen

**Figure 1:**
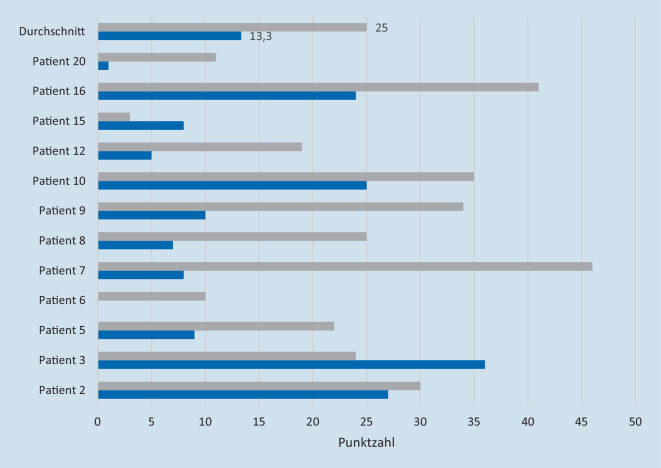

Die Beantwortung des Fragebogens mit den jeweiligen Angehörigen der Patienten konnte in 12 Fällen erreicht werden. Der errechnete Mittelwert der bei der Angehörigenbefragung erreichten Punktzahl betrug 25 Punkte (Spannbreite von 3 bis 46 Punkten; Tab. 3). In 84 % der Fälle ergab die Befragung der Angehörigen einen höheren Score; nur bei 3 der 12 Patienten-Angehörige-Paare (16 %) vergaben die Angehörigen eine niedrigere Punktezahl (Abb. 1). Im Durchschnitt vergaben die Angehörigen der Patienten einen um 11,7 Punkte höheren Gesamtpunktewert als die Patienten selbst.

Diese Unterschiede der Selbstreflexion können durch Beispiele illustriert werden. Patient Nummer 6 beantwortete in der MBI‑C sämtliche Fragen mit 0 Punkten, seine Ehefrau hingegen gab ihm insgesamt 10 Punkte (der Patient entließ sich letztendlich gegen ärztlichen Rat). Patientin Nummer 8 gab zwar an, jemand wolle sie bestehlen, vergab bei der entsprechenden Frage der MBI‑C jedoch eine kleinere Punktzahl als ihre Tochter, die von „Halluzinationen“ sprach.

## Diskussion

Im Rahmen des hier vorgestellten Projekts wurde erstmals eine deutsche Übersetzung der MBI‑C erstellt, auf Verständlichkeit und Anwendbarkeit geprüft und schließlich veröffentlicht. Die MBI‑C kann unter www.mbitest.org kostenlos heruntergeladen werden.

Die MBI‑C kann selbstständig durch die untersuchte Person beantwortet werden. Dies ist v. a. dann ausreichend, wenn das Instrument im Rahmen eines primärprophylaktischen Screenings bei Personen ohne signifikante neuropsychiatrische Defizite angewendet wird.

In dieser Studie wurde die MBI‑C in Form eines Interviews mit den stationär behandelten Patienten sowie mit ihren Familienangehörigen angewendet.

Die Bereitschaft der Patienten und deren Angehörigen zur Beantwortung des Fragebogens sowie die Verständlichkeit der gestellten Fragen zeigten sich insgesamt als sehr gut. Der Zeitaufwand für die Beantwortung der Fragen betrug bei 76 % der Patienten zwischen 10 und 20 min. Der Mittelwert lag bei 16 min.

Die gewählte Patientengruppe stellt besonders hohe Anforderungen an die Anwendbarkeit. Wir gehen davon aus, dass bei der primären Zielgruppe für die Anwendung der Skala – ambulanten Patienten mit nur leichten Symptomen – die Akzeptanz noch höher und der Zeitaufwand geringer sein dürften.

Bei Befragung der Patienten (*n* = 21) wurden durchschnittlich 13 von maximal 102 Punkten erreicht. Bei Befragung von Familienangehörigen (*n* = 12) wurden durchschnittlich 25 Punkte erreicht.

Der Unterschied der Wahrnehmung des Vorliegens der neuropsychiatrischen Symptome zwischen den Patienten mit MBI und deren Familienangehörigen wurde bereits bei der Anwendung der englischen Originalvariante der MBI‑C beschrieben [5]. Die simultane Anwendung der MBI‑C bei Patienten und deren Angehörigen kann eine strukturierte Untersuchung dieses Phänomens ermöglichen. Gleichzeitig wird durch die Befragung der Angehörigen die Sensitivität des Instruments erhöht. Aus diesen Gründen empfehlen wir auch bei zukünftiger Anwendung der MBI‑C, nach Möglichkeit sowohl Patienten als auch Angehörige bzw. Bezugspersonen zu befragen.

Das MBI-Konzept soll primär bei der Verbesserung des Erkennens eines Frühstadiums neurodegenerativer Demenzen behilflich sein. Für diese Aufgabe ist auch die MBI‑C adaptiert. Bei Patienten mit einer bereits vorliegenden Demenz ist definitionsgemäß die Diagnose eines MBI nicht mehr möglich. Hier unterscheidet sich die MBI‑C von dem breit etablierten NPI-Questionnaire (NPI‑Q; [6]), welcher häufig zur Erfassung der NPS bei Patienten mit Demenz dient. Auch durch die Anwendung direkt bei den untersuchten Personen, und nicht nur bei den Bezugspersonen, unterscheidet sich die MBI‑C vom NPI‑Q.

Bei der Studienpopulation lagen häufig depressive Symptome vor. So wurde bei 9 von 19 Patienten, bei welchen der GDS-15-Fragebogen beantwortet wurde, eine Punktzahl > 5 erreicht, was das Vorliegen einer Depression impliziert (Tab. 3). Ein Zusammenhang von depressiven Symptomen mit der Frühentwicklung einer neurodegenerativen Demenzerkrankung wird häufig diskutiert. Um die Korrelation einzelner depressiver und anderer neuropsychiatrischer Symptome mit der Entwicklung einer neurodegenerativen Demenz zu untersuchen, werden prospektive Untersuchungen über einen Zeitraum von mehreren Jahren notwendig sein.

Mit anderen Sprachmutationen der MBI‑C wurden solche Studien bereits durchgeführt. So konnte eine Korrelation zwischen dem Vorliegen eines MBI bei kognitiv gesunden Probanden und einem beschleunigten kognitiven Abbau in einem Beobachtungszeitraum von einem Jahr nachgewiesen werden [4]. Außerdem zeigte sich eine Assoziation zwischen dem Vorliegen eines MBI und dem Nachweis von verschiedenen Biomarkern einer neurodegenerativen Erkrankung [13, 16]. Neben dem Konzept eines MCI erscheint somit auch das MBI-Konzept als hilfreich bei der frühzeitigen Identifikation von Personen mit erhöhtem Risiko der Entwicklung einer neurodegenerativen Demenz.

### Limitationen

Die Limitationen bei der Anwendung der Checkliste hängen v. a. mit der Auswertung des Instruments zusammen. Die MBI‑C soll bei dem Festlegen des Vorliegens eines MBI helfen. In verschiedenen Studien werden aber unterschiedliche Cut-off-Werte angewendet [3, 14]. Der hier gezeigte Unterschied in der Bewertung durch die Patienten und ihre Familienangehörige zeigt zusätzlich, dass auch dieser Aspekt in der Datenauswertung beachtet werden muss. Ebenfalls ist bisher unbekannt, ob einzelne Symptome oder Symptombereiche bei der Beurteilung des Risikos einer demenziellen Entwicklung ggf. sensitiver sind als die anderen. Schließlich ist auch die Wichtigkeit der Beschreibung der Intensität der Symptome noch unbekannt. Dank der Anwendung der standardisierten Checkliste können aber gerade solche Fragen zukünftig präziser beurteilt und beantwortet werden.

## Fazit für die Praxis

- Mit der MBI‑C liegt ein Instrument vor, welches eine strukturierte Untersuchung der neuropsychiatrischen Symptome bei älteren Personen erleichtert.
- Die hier vorgestellte deutschsprachige Version ermöglicht es nun, auch deutschsprachige Patienten in prospektive klinische Studien zur Erforschung des MBI zu rekrutieren, um dadurch den Stellenwert dieses Syndroms zukünftig besser zu verstehen.
- Auch eine studienunabhängige Anwendung der MBI‑C in der klinischen Routine kann helfen, die beurteilten Symptome bei den Patienten frühzeitig zu erkennen und deren Präsenz in den diagnostischen sowie therapeutischen Maßnahmen abzubilden.

### Supplementary Information




