# Supplementary material for: Mild behavioral impairment checklist: English-German translation and feasibility study assessing its use in clinical practice
Source: Z Gerontol Geriatr. 2023 Jun 26;57(3):207–13. [Article in German] doi: 10.1007/s00391-023-02200-4 (PMC11078794; doi:10.1007/s00391-023-02200-4)
Supplement: Supplementary file 1 [file 391_2023_2200_MOESM1_ESM.docx]

| **Klinische Diagnosen** | **Anzahl** |
| --- | --- |
| Bipolar affektive Störung (F31.4) | 2 |
| Mittelgradige depressive Episode (F32.1) | 5 |
| Schwere depressive Episode ohne psychotische Symptome (F32.2) | 6 |
| Schwere depressive Episode mit psychotischen Symptomen (F32.3) | 2 |
| Rezidivierende depressive Störung, gegenwärtig mittelgradige Episode (F33.1) | 2 |
| Rezidivierende depressive Störung, gegenwärtig schwere Episode ohne psychotische Symptome (F33.2) | 1 |
| Rezidivierende depressive Störung, gegenwärtig schwere Episode mit psychotischen Symptomen (F33.3) | 1 |
| Panikstörung (episodisch paroxysmale Angst; F41.0) | 4 |
| Generalisierte Angststörung (F41.1) | 1 |
| Undifferenzierte Somatisierungsstörung (F45.1) | 3 |

**Supplement** **Tabelle 1** Klinisch gestellte Diagnosen der Studienpatienten (n=21). Die Entlassungsdiagnosen wurden vom klinischen Behandlungsteam unabhängig von der Beurteilung der Patienten im Rahmen der Studie gestellt. Bei einem Patienten sind mehrere Diagnosen gleichzeitig möglich. In Klammern Angabe des ICD-10 Codes.
